# Commentary on article by Ferguson et al.: “A comparison of lateral release rates in fixed- versus mobile-bearing total knee arthroplasty”

**DOI:** 10.1007/s10195-015-0355-x

**Published:** 2015-05-19

**Authors:** Matthew P. Abdel

**Affiliations:** Department of Orthopedic Surgery, Mayo Clinic, 200 First Street S.W., Rochester, MN 55905 USA

Many surgeons have been intrigued by mobile-bearing (MB) total knee arthroplasty (TKA) designs due to the concept of self-alignment and the suggestion that those designs can accommodate small mismatches in the position of the femoral and tibial components. The natural question of course is whether this self-alignment translates into improved patellar tracking, thus decreasing the prevalence of lateral retinacular releases. In any medical or surgical specialty, the backbone of the literature is randomized clinical trials (RCTs). In this excellent RCT completed by Ferguson et al. [1], the authors randomized 352 patients undergoing TKA to receive either a MB or fixed-bearing (FB) construct. In addition, patients were further sub-randomized into patellar resurfacing or retention groups.

As shown in other RCTs [3, 4], the authors found a similar lateral release rate between the FB and MB groups. However, the rate in both groups (approximately 10 %) was higher than most contemporary reports [4]. Of note, there were two additional interesting findings. First, patellar resurfacing resulted in significant lower release rates (6 % vs. 14 %, respectively; *P* = 0.0179). Second, the lower release rate in resurfaced patellae was significant only when a MB construct was utilized. In essence, it appears that the use of a MB construct and resurfacing the patella are symbiotic, resulting in a 3 % lateral release rate. In comparison, there was a 16 % lateral release rate for retained patellae in the MB group, and an 11 % lateral release rate for retained patellae in the FB group.

This study has several strengths. Foremost, it is a well-executed RCT with a single knee design (i.e., DePuy P.F.C. Sigma^®^), an identical fixation method (i.e., cemented), and a similar postoperative rehabilitation course in both groups. In addition, it appears that no patients withdrew from the study or were lost to follow-up. Unfortunately, a power analysis was not included. A power analysis not only helps the reader know the sample size required to detect an effect of a given size with a given degree of confidence, but also tells the reader what outcome parameter [i.e., lateral release rates, Knee Society Scores (KSS), anterior knee pain, etc.] the power analysis was completed to investigate.

In 2004, Pagnano et al. [4] from the Mayo Clinic completed a RCT of 240 patients randomized to an all-polyethylene tibia, a modular metal-backed tibia, or a rotating-platform (RP) tibia with the same femoral component. The authors found no difference in lateral release rates between any groups, with the prevalence being 3.8 % in each group. However, the prevalence of patellar tilt was 5 % in the all-polyethylene group, 7 % in the modular metal-backed group, and 11 % in the RP group. In an update of a 2015 Cochrane review on cruciate-retaining TKAs, no difference was noted between FB and MB in regards to knee pain, clinical and functional scores, health-related quality of life, revision surgery, mortality, reoperation rate, and other serious adverse events [2]. Similarly, Smith et al. [5] completed a meta-analysis and systematic review of 14 RCTs and found no difference between MB and FB TKAs in regards to KSS, Hospital for Special Surgery (HSS) scores, or range of motion.

In summary, this study has added to the growing body of literature suggesting no significant difference between MB and FB TKAs, particularly with regards to lateral release rates.
